# Transcriptomic Analysis of the Activity of a Novel Polymyxin against *Staphylococcus aureus*

**DOI:** 10.1128/mSphere.00119-16

**Published:** 2016-07-27

**Authors:** Jinxin Zhao, Soon-Ee Cheah, Kade D. Roberts, Roger L. Nation, Philip E. Thompson, Tony Velkov, Zongjun Du, Matthew D. Johnson, Jian Li

**Affiliations:** a College of Marine Science, Shandong University at Weihai, Weihai, China; b Drug Delivery, Disposition and Dynamics, Monash Institute of Pharmaceutical Sciences, Monash University, Victoria, Australia; c Medicinal Chemistry, Monash Institute of Pharmaceutical Sciences, Monash University, Victoria, Australia; d Monash Biomedicine Discovery Institute, Department of Microbiology, Monash University, Victoria, Australia; University of Nebraska Medical Center

**Keywords:** *Staphylococcus aureus*, gene expression, polymyxins

## Abstract

Polymyxin B and colistin are exclusively active against Gram-negative pathogens and have been used in the clinic as a last-line therapy. In this study, we investigated the antimicrobial activity of a novel polymyxin, FADDI-019, against *Staphylococcus aureus*. MIC and time-kill assays were employed to measure the activity of FADDI-019 against *S. aureus* ATCC 700699. Cell morphology was examined with scanning electron microscopy (SEM), and cell membrane polarity was measured using flow cytometry. Transcriptome changes caused by FADDI-019 treatment were investigated using transcriptome sequencing (RNA-Seq). Pathway analysis was conducted to examine the mechanism of the antibacterial activity of FADDI-019 and to rationally design a synergistic combination. Polymyxin B and colistin were not active against *S. aureus* strains with MICs of >128 mg/liter; however, FADDI-019 had a MIC of 16 mg/liter. Time-kill assays revealed that no *S. aureus* regrowth was observed after 24 h at 2× to 4× MIC of FADDI-019. Scanning electron microscopy (SEM) and flow cytometry results indicated that FADDI-019 treatment had no effect on cell morphology but caused membrane depolarization. The vancomycin resistance genes *vraRS*, as well as the VraRS regulon, were activated by FADDI-019. Virulence determinants controlled by SaeRS and the expression of enterotoxin genes *yent2*, *sei*, *sem*, and *seo* were significantly downregulated by FADDI-019. Pathway analysis of transcriptomic data was predictive of a synergistic combination comprising FADDI-019 and sulfamethoxazole. Our study is the first to examine the mechanism of the killing of a novel polymyxin against *S. aureus*. We also show the potential of transcriptomic and pathway analysis as tools to design synergistic antibiotic combinations.

**IMPORTANCE** *S. aureus* is currently one of the most pervasive multidrug-resistant pathogens and commonly causes nosocomial infections. Clinicians are faced with a dwindling armamentarium to treat infections caused by *S. aureus*, as resistance develops to current antibiotics. This accentuates the urgent need for antimicrobial drug discovery. In the present study, we characterized the global gene expression profile of *S. aureus* treated with FADDI-019, a novel synthetic polymyxin analogue. In contrast to the concentration-dependent killing and rapid regrowth in Gram-negative bacteria treated with polymyxin B and colistin, FADDI-019 killed *S. aureus* progressively without regrowth at 24 h. Notably, FADDI-019 activated several vancomycin resistance genes and significantly downregulated the expression of a number of virulence determinants and enterotoxin genes. A synergistic combination with sulfamethoxazole was predicted by pathway analysis and demonstrated experimentally. This is the first study revealing the transcriptomics of *S. aureus* treated with a novel synthetic polymyxin analog.

## Abstract (precis)

*S. aureus* is currently one of the most pervasive multidrug-resistant pathogens and commonly causes nosocomial infections. Clinicians are faced with a dwindling armamentarium to treat infections caused by *S. aureus*, as resistance develops to current antibiotics. This accentuates the urgent need for antimicrobial drug discovery. In the present study, we characterized the global gene expression profile of *S. aureus* treated with FADDI-019, a novel synthetic polymyxin analogue. In contrast to the concentration-dependent killing and rapid regrowth in Gram-negative bacteria treated with polymyxin B and colistin, FADDI-019 killed *S. aureus* progressively without regrowth at 24 h. Notably, FADDI-019 activated several vancomycin resistance genes and significantly downregulated the expression of a number of virulence determinants and enterotoxin genes. A synergistic combination with sulfamethoxazole was predicted by pathway analysis and demonstrated experimentally. This is the first study revealing the transcriptomics of *S. aureus* treated with a novel synthetic polymyxin analog.

## INTRODUCTION

Polymyxin B and colistin (i.e., polymyxin E) are naturally occurring cationic antimicrobial peptides (CAMPs) that have been used in the clinic for over 50 years to treat multidrug-resistant Gram-negative bacterial infections (1–3). Polymyxins primarily interact with lipid A, which is located in the outer leaflet of the Gram-negative outer membrane (OM) (1, 3, 4). It has been reported that CAMPs interact with cell membranes and form membrane-spanning pores, which results in cell death (5, 6). In addition to causing membrane perturbation, CAMPs can also inhibit intracellular metabolic processes, including biosynthesis of the cell wall, nucleic acids, and proteins (7, 8). In these cases, the cell death may be the result of multiple inhibitory effects. It has been shown that the interaction of polymyxins with lipid A is essential to their antimicrobial activity in Gram-negative bacteria (9). Gram-positive bacteria lack an OM or lipid A, rendering them inherently resistant to polymyxin B and colistin. Interestingly, we have shown that novel polymyxins can have very different activities against both Gram-negative and Gram-positive pathogens (3, 9). Specifically, FADDI-019 is a novel polymyxin B derivative with a d-octylglycine at position 6 and an octanoyl fatty acyl chain at the N terminus, as opposed to polymyxin B1 and colistin A, which have either a d-phenylalanine (polymyxin B1) or d-leucine (colistin A) at position 6 and a 6-*S*-methlyoctanoyl fatty acyl chain (9). With increased hydrophobicity within its heptapeptide ring, FADDI-019 exhibits increased activity against Gram-positive bacteria, including *Staphylococcus aureus* (9). More recently, other polymyxin-based synthetic CAMPs have been designed that exhibit antimicrobial activities against Gram-positive bacteria (10, 11). Synthetic CAMPs containing tryptophan, arginine, and *N-*leucine alterations showed greater activities against Gram-positive bacteria; in addition, these CAMPs showed low cytotoxicities and activities against Gram-negative bacteria, equivalent to those of polymyxin B (10, 12). Gallardo-Godoy et al. generated a library of synthetic polymyxin B derivatives, four of which showed significantly reduced MICs against *S. aureus* (11). Despite the growing number of synthetic polymyxin-like CAMPs, there is currently no understanding of the transcriptional changes caused by their activity against Gram-positive bacteria.

Many Gram-negative species use well-characterized two-component systems to develop resistance to polymyxin (13, 14). In *Salmonella enterica* serotype Typhimurium, polymyxins activate the two-component system PhoPQ, causing the activation of the PhoPQ regulon (13, 15). The *pmr* (polymyxin resistance) locus is part of that regulon, and the activation of the *pmr* locus results in modifications of lipid A, which in turn serve to decrease the interaction with polymyxins. This mechanism has also been shown in *Pseudomonas aeruginosa*, *Escherichia coli*, and *Klebsiella pneumoniae* (16–20). Due to the inactivity of polymyxin B and colistin against Gram-positive bacteria, the transcriptional response of Gram-positive bacteria to polymyxins has not been investigated. The transcriptional responses to CAMPs other than polymyxins have been reported in *S. aureus* and *Bacillus subtilis* (21, 22). Pietiäinen et al. characterized the transcriptional response of *S. aureus* to three CAMPs, ovisporin-1, temporin L, and dermaseptin K4-S4 (22). Significant activation of the *vraSR* and *vraDE* genes, which are involved in vancomycin resistance and cell wall homeostasis, suggested that these CAMPs perturb the cell wall. In *B. subtilis*, treatment with CAMPs resulted in the activation of the SigW and SigM cytoplasmic sigma factors and the YxdJK and LiaRS two-component systems (21). In scenarios where antimicrobials target multiple components of the bacterial cell, transcriptome analysis is capable of identifying affected pathways (21–24). In the present study, we characterized the response of *S. aureus* ATCC 700699 treated with a novel synthetic polymyxin, FADDI-019, using phenotypic assays and transcriptomics. Furthermore, we successfully predicted a synergistic combination by targeting a choke point identified using pathway analysis of our transcriptomic data.

## RESULTS

### Antimicrobial activity of FADDI-019 against *S. aureus*.

In order to characterize the antimicrobial activities of FADDI-019, the MICs of FADDI-019 were measured against *S. aureus* ATCC 700699 (Mu50), *S. aureus* ATCC 700698 (Mu3), *P. aeruginosa* ATCC 27853, and *Acinetobacter baumannii* ATCC 19606 (Table 1). The MICs of FADDI-019 against *S. aureus* strains ATCC 700699 and ATCC 700698 were both 16 mg/liter, consistent with our previous report (9). We also tested polymyxin B and colistin against both *S. aureus* strains and observed no activity (MICs of >128 mg/liter) for both compounds, confirming that these *S. aureus* strains are intrinsically resistant to the naturally occurring, clinically available polymyxins. The results in Table 1 show that FADDI-019 had MICs between 1 and 2 mg/liter against Gram-negative bacteria. This level of activity is comparable to those of polymyxin B and colistin against the same strains (Table 1). In summary, FADDI-019 displayed antibacterial activity against *S. aureus* strains that were resistant to polymyxin B and colistin.

**TABLE 1:** MICs of polymyxin B, colistin, and FADDI-019 against Gram-positive and Gram-negative strains

Strain	MIC (mg/liter) of:		
	Polymyxin B	Colistin	FADDI-019
*A. baumannii* ATCC 19606	0.5	1	1
*A. baumannii* ATCC 17978	2	0.5	2
*P. aeruginosa* ATCC 27853	2	2	2
*S. aureus* ATCC 700698	>128	>128	16
*S. aureus* ATCC 700699	>128	>128	16

### FADDI-019 killing kinetics against *S. aureus* and SEM analysis of cell morphology.

**FIG 1:**
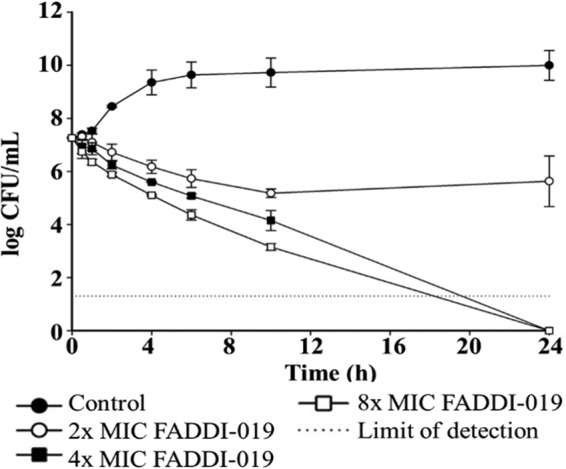
Time-kill kinetics of FADDI-019 against *S. aureus* ATCC 700699. The error bars show the standard deviations of the results from three independent biological repeats.

**FIG 2:**
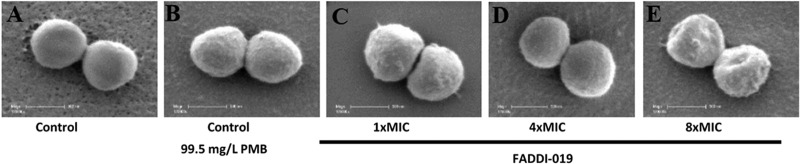
SEM images of *S. aureus* ATCC 700699 cells left untreated (A) or treated with polymyxin B (PMB; 99.5 mg/liter) (B), FADDI-019 (1× MIC) (C), FADDI-019 (4× MIC) (D), and FADDI-019 (8× MIC) (E) for 1 h. The white scale bar in each figure represents 500 nm.

Figure 1 shows the killing kinetics of FADDI-019 at three concentrations (i.e., 2×, 4×, and 8× MIC) against *S. aureus* strain ATCC 700699. Contrary to the typical rapid-killing kinetics of polymyxin B and colistin against Gram-negative bacteria (25–28), FADDI-019 caused slow killing against *S. aureus* ATCC 700699 at all concentrations tested; interestingly, regrowth was not observed even at 2× MIC. Scanning electron microscopy (SEM) was used to investigate the effects of FADDI-019 treatment on cell morphology and the bacterial cell surface of *S. aureus* ATCC 700699 (Fig. 2). Treatment with polymyxin B (99.5 mg/liter) was used for comparison, which showed no significant morphological alterations of *S. aureus* ATCC 700699 cells. Interestingly, compared to the results for the untreated control, no major perturbations of cell morphology were observed in the treated samples at 1× or 4× MIC of FADDI-019, even with significant bacterial killing (Fig. 1). Cell morphology changes were only evident at 8× MIC of FADDI-019 against *S. aureus* ATCC 700699.

### Flow cytometry analysis of the membrane polarity during FADDI-019 treatment.

**FIG 3:**
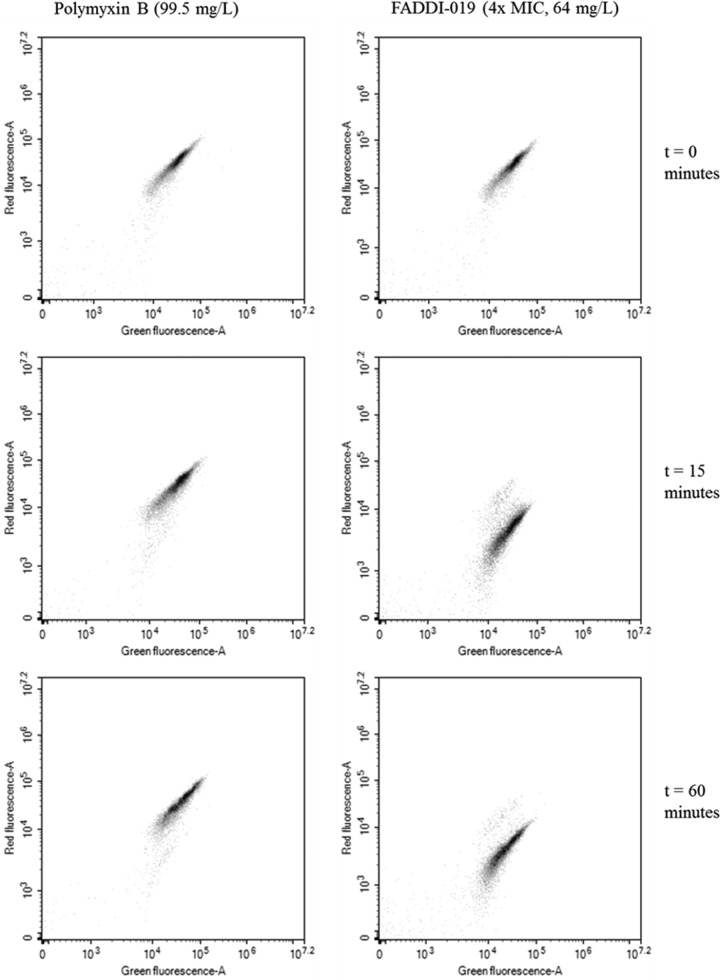
Scatter plots of flow cytometry analysis of *S. aureus* ATCC 70099 during treatment with polymyxin B and FADDI-019. DiOC_2_(3) was used to stain the cells, and the fluorescence of each incident (indicated as a dot) in the red and green channels was measured and plotted onto a 2-D scatterplot. Shifting of the scatter in the red fluorescence channel is indicative of membrane depolarization.

*S. aureus* cells treated with polymyxin B at 99.5 mg/liter for up to 1 h showed no indication of depolarization relative to the results for the control at time zero (Fig. 3). Interestingly, there was a significant shift in the red fluorescence when cells were treated with FADDI-019 at 4× MIC within 15 min, indicating that treatment with FADDI-019 resulted in membrane depolarization (Fig. 3). In addition, there was no evidence of recovery from membrane depolarization after 60 min of treatment (Fig. 3), which suggests that the depolarization effect of FADDI-019 was permanent.

### Transcriptome profiling and enrichment of *S. aureus* ATCC 700699 in response to FADDI-019 treatment.

**FIG 4:**
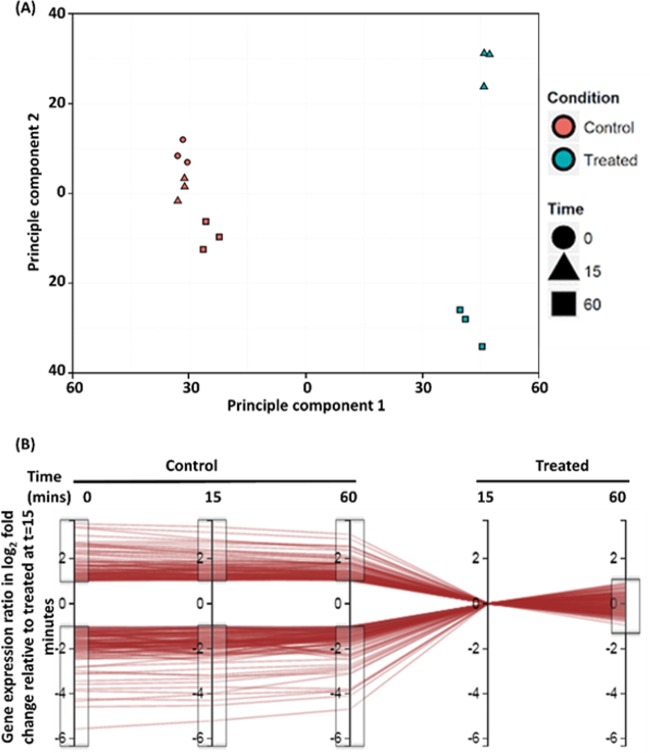
(A) PCA results for all data, with individual samples plotted on principal component 1 (horizontal axis) and principal component 2 (vertical axis). (B) Graphical representation of the gene enrichment process using Degust. Individual differentially regulated genes are represented as horizontal red lines. The vertical axes represent the log_2_ fold change of all genes at each time point in control and treated conditions (indicated above each axis) relative to 15 min of treatment with FADDI-019. Gray boxes indicate the gating cutoffs used to enrich the gene list for genes that are greater than 1 log_2_ fold differentially expressed throughout the control samples but >1 log_2_ fold differentially expressed after 60 min.

The transcriptome variances of all samples, treatments, and time points were analyzed by principal component analysis (PCA) (Fig. 4A). PCA analysis separated FADDI-019-treated from untreated samples across principal component 1, indicating that treatment with FADDI-019 was responsible for 51.5% of total variance across all samples (Fig. 4A). Samples taken at different time points were separated across principal component 2, which was responsible for 14.9% of the total variance between all samples (Fig. 4A). Individual repeats clustered together, showing the excellent reproducibility of our transcriptomic study (Fig. 4A). Voom and limma linear modeling and Degust (Fig. 4B) were used to enrich for genes that were differentially regulated at 15 min and sustained this response after 60 min of FADDI-019 treatment (false discovery rate [FDR] = 0.05, >2-fold change) (Fig. 4B). After this enrichment, a total of 208 differentially regulated genes were identified, of which 140 were significantly upregulated and 68 significantly downregulated (FDR = 0.05, >2-fold change) (Fig. 4B). The use of a time course allowed enrichment analysis, which limited the list of differentially regulated genes to those involved in a specific response to FADDI-019 (see Table S1 in the supplemental material).

10.1128/mSphere.00119-16.1 Table S1  Full list of differentially regulated genes and their respective expression ratios, expressed in log_2_, false discovery rate (FDR) values, annotations, and pathways. Download Table S1, PDF file, 0.4 MB. Copyright © 2016 Zhao et al. 2016 Zhao et al. This content is distributed under the terms of the Creative Commons Attribution 4.0 International license.

### Induction of the vancomycin resistance regulon in *S. aureus* ATCC 700699 by FADDI-019.

**FIG 5:**
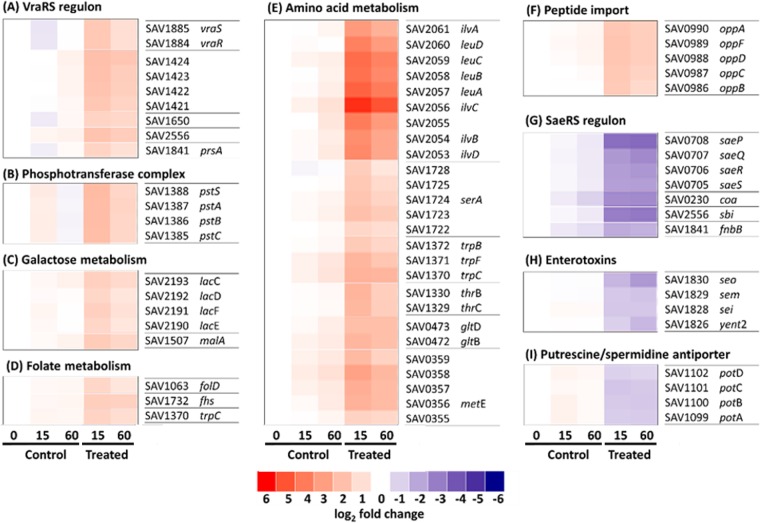
(A to I) Heat map representation of gene expression relative to that in the control at time zero. Gene numbers and annotations are displayed adjacent to each map. Red-to-blue colors represent the log_2_ fold change, ranging from 6(log_2_)-fold upregulated (red) through no change (white) to −6(log_2_)-fold change (blue). Each map was generated using data from three biological repeats, and all genes represented have a >2-fold change in expression after 15 min of treatment relative to their expression in control samples.

Genes involved in similar cellular processes are often regulated in kind (29). We analyzed the enriched gene lists to identify the pathways affected by FADDI-019 treatment. Analysis of genes upregulated by FADDI-019 in *S. aureus* ATCC 700699 showed a significant increase in the expression of the vancomycin resistance genes. Constituents of the two-component signaling system comprising *vraRS* were upregulated by FADDI-019 5.04- and 4.90-fold, respectively (Fig. 5A). In order to confirm the activation of the remaining VraSR regulon, we analyzed the expression of the genes known to be under the control of the VraSR two-component system. These included hypothetical proteins SAV1424, SAV1423, SAV1422, SAV1421, and SAV2556, which were upregulated 3.50-, 3.90-, 3.73-, 3.50-, and 4.28-fold, respectively, hypothetical membrane protein SAV1650, which was upregulated 3.54-fold, and a peptidyl-prolyl isomerase, *prsA*, which was upregulated 3.41-fold (Fig. 5A). These results show that, in addition to the activation of VraSR, genes in the VraSR regulon were also upregulated during FADDI-019 treatment.

### Activation of genes involved in the metabolism of energy, folate, and amino acids by FADDI-019.

FADDI-019-treated *S. aureus* ATCC 700699 showed an increase in the expression of genes involved in basic metabolic processes, including peptide import, metabolism of folate and sugar, and amino acid biosynthesis. The phosphotransferase system genes *pstSACB*, which code for proteins involved in sugar import, were upregulated 2.08-, 2.88-, 2.88-, and 3.57-fold, respectively (Fig. 5B). In addition, the galactose metabolism genes *lacCDEF* and the *malA* gene were upregulated 2.37-, 2.18-, 2.27-, 2.09-, and 2.79-fold, correspondingly (Fig. 5C). Genes involved in folate biosynthesis, *fhs* and *folD*, were also upregulated 2.14- and 2.47-fold, respectively (Fig. 5D). The most-upregulated genes during FADDI-019 treatment were those involved in amino acid biosynthesis, and the levels of gene expression ranged from 13.12- to 23.07-fold higher than in the control (Fig. 5E). In addition to amino acid biosynthesis, peptide import genes were also significantly upregulated. The *oppABCDF* locus encodes a peptide import system, and genes in this operon were 3.11- to 3.31-fold upregulated in FADDI-019-treated cells relative to their expression in the control (Fig. 5F).

### Repression of key virulence factors in *S. aureus* ATCC 700699 treated with FADDI-019.

Of the 208 genes that were differentially expressed during the FADDI-019 treatment, 68 were downregulated. The main pathways repressed by FADDI-019 treatment are involved in virulence. The *saeSR* genes, which code for the SaeSR two-component system, were downregulated −3.89-fold and −4.03-fold, respectively (Fig. 5G). The SaeSR two-component system is responsible for the activation of numerous *S. aureus* virulence genes, including *coa*, *sbi*, *fnbB*, *saeQ*, and *saeP* (Fig. 5G) (30–35). The *coa*, *sbi*, and *fnbB* genes, which code for host cell-associated proteins, were downregulated −4.49-fold, −6.83-fold, and −2.58-fold, correspondingly (Fig. 5G). The *saeQ* and *saeP* genes, which encode regulatory proteins that increase the phosphatase activity of SaeS, were repressed −4.19-fold and −8.88-fold, respectively (Fig. 5G). In addition to the repression of virulence pathways, FADDI-019 treatment also had significant effects on known pathogenicity determinants in *S. aureus*. Four enterotoxin genes, *yent2*, *sei*, *sem*, and *seo*, which encode proteins that are the causative agents of toxic shock, were downregulated −2.09-fold, −2.67-fold, −2.19-fold, and −2.94-fold, respectively (Fig. 5H) (36). The import of polyamines has been linked to a number of processes, including signaling and infection (37, 38). The putrescine/spermidine importer *potABCD* was repressed by FADDI-019 treatment. The *potABCD* genes were significantly downregulated, by −2.85-fold, −2.96-fold, −2.80-fold, and −2.56-fold, respectively, relative to their expression in the untreated control (Fig. 5I).

### Identification of a folate metabolism chokepoint.

**FIG 6:**
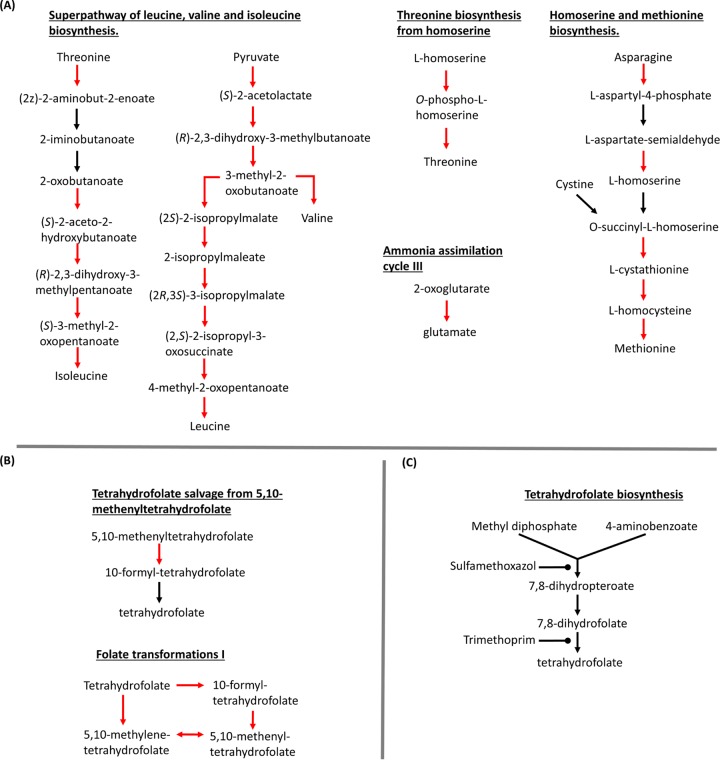
(A) Metabolic pathways of the amino acid biosynthesis affected by FADDI-019 treatment. (B) Tetrahydrofolate pathway affected by FADDI-019 treatment. (C) Tetrahydrofolate biosynthesis pathway targeted by sulfamethoxazole and trimethoprim. Arrows indicate reactions catalyzed by known enzymes in *S. aureus* 700698, and red arrows indicate reactions catalyzed by enzymes with >2-fold increased expression.

We used a systems biology approach to identify metabolic chokepoints based on the transcriptomic response to FADDI-019 treatment. Pathway analysis using the transcriptome data identified that each amino acid biosynthesis system was overexpressed (Fig. 6A). Importantly, the folate metabolic pathways were also overexpressed (Fig. 6B). Tetrahydrofolate is a key metabolite for amino acid biosynthesis (39); our pathway analysis indicated that amino acid metabolism is dependent on folate metabolism and that the tetrahydrofolate synthesis pathway is a suitable chokepoint to inhibit both processes. Sulfamethoxazole is an antimicrobial that inhibits the tetrahydrofolate metabolic pathway (Fig. 5C) (40–42). We measured the MICs of sulfamethoxazole in combination with FADDI-019 against *S. aureus*. Table 2 shows the MICs of FADDI-019 and sulfamethoxazole against *S. aureus* ATCC 700699. Sulfamethoxazole alone had a MIC greater than 128 mg/liter, indicating that it had no antimicrobial activity against *S. aureus* ATCC 700699. In combination, 16 mg/liter sulfamethoxazole decreased the FADDI-019 MIC substantially, to 4 mg/liter, against *S. aureus* ATCC 700699.

**TABLE 2:** MICs of FADDI-019 and sulfamethoxazole against Gram-positive strains

Strain	MIC (mg/liter) of:		
	FADDI-019	SMX^a^	FADDI-019 + SMX
*S. aureus* ATCC 700698	16	>128	4/16
*S. aureus* ATCC 700699	16	>128	4/16

a SMX, sulfamethoxazole.

## DISCUSSION

Polymyxins have been used as a last-line therapeutic option against Gram-negative pathogens (43–45). However, as their mode of action requires the initial binding to lipid A in the outer membrane, polymyxin B and colistin are inactive against Gram-positive bacteria (3, 46). Furthermore, resistance to polymyxins is frequently acquired *in vitro* within 24 h of treatment by loss or modification of lipid A (16, 17, 47–50). Previously, we have shown that a novel polymyxin, FADDI-019, has antimicrobial activity against Gram-positive bacteria (3, 9). FADDI-019 was designed with a major alteration from the clinically available polymyxin B and colistin. FADDI-019 has a d-octylglycine at position 6, whereas polymyxin B and colistin have d-phenylalanine and d-leucine residues, respectively, at this location (3, 9). Hence, the hydrophobicity of FADDI-019 at position 6 is greater than that of polymyxin B or colistin. The predicted effect of this difference is to increase the hydrophobic reach of the molecule at position 6. Previously, we have shown that both polar and hydrophobic residues are important for the action of polymyxins against Gram-negative bacteria (3). The activity of FADDI-019 against Gram-positive bacteria indicated that increased hydrophobicity is crucial for the antimicrobial activity of the core polymyxin structure against Gram-positive bacteria. It is evident that FADDI-019 has a wider antimicrobial spectrum than do polymyxin B and colistin (3). Time-kill assays revealed that no regrowth of *S. aureus* ATCC 700699 was observed after treatment, which is in contrast to the killing of polymyxins against Gram-negative bacteria, where regrowth of polymyxin-resistant variants is frequently observed even within 12 h (26, 27). In addition to the aforementioned properties of FADDI-019, we have previously reported that, in rodent models, FADDI-019 has a tolerability equivalent to that of colistin and polymyxin B after intravenous (0.75 mg/kg of body weight) and subcutaneous (40 mg/kg) administration (3). FADDI-019 also lacks hemolytic activity, similar to polymyxin B and colistin. However, it should be noted that the MIC of FADDI-019 against *S. aureus* is between 4- and 8-fold higher than that of polymyxin B or colistin against Gram-negative bacteria (26, 51) and that this may limit the use of FADDI-019 as a therapeutic.

The analysis of cell morphology by SEM after treatment revealed that cell blebbing was not induced by FADDI-019 at 1× or 4× MIC, although morphology changes were evident at 8× MIC. Membrane blebbing is a common feature of polymyxin-treated Gram-negative bacteria (52, 53), so the absence of membrane blebbing is suggestive of an alternative killing mechanism. To further investigate the effect of FADDI-019 treatment, we examined the membrane polarity of *S. aureus* ATCC 700699 cells by flow cytometry. FADDI-019 depolarized the cell membrane within 15 min, indicating that, despite the lack of membrane blebbing, FADDI-019 interacted with the *S. aureus* cell membrane. Our results show that the antimicrobial mechanism of FADDI-019 against *S. aureus* is different from previously characterized polymyxin mechanisms against Gram-negative pathogens (5). Furthermore, the lack of lipid A, absence of membrane blebbing, and extensive membrane depolarization are suggestive of an alternative target of FADDI-019.

Analysis of transcriptomic changes caused by FADDI-019 treatment showed that a number of major pathways associated with vancomycin resistance were upregulated. The *vraSR* two-component system, which was originally characterized for its role in vancomycin resistance, is responsible for coordinating the response to antimicrobials that target the cell wall (54). Transcriptomic studies that assayed the effects of ovisporin-1, temporin L, and dermaseptin K4-S4 against *S. aureus* ATCC 25904 have been reported previously (21, 22). Unlike the cyclic FADDI-019, ovisporin-1, temporin L, and dermaseptin K4-S4 are all linear alpha-helical CAMPs. The upregulation of vancomycin resistance genes was also observed with alpha-helical CAMP treatments against *S. aureus* (22), which suggests that cell wall homeostasis has a key role in the response to all CAMPs that show antimicrobial activity against Gram-positive bacteria. Amino acid biosynthesis genes and peptide import systems were also upregulated in response to alpha-helical CAMP treatments (21). The predominant amino acid biosynthesis pathway upregulated by FADDI-019 treatment was the leucine, isoleucine, and valine superpathway. Components of other amino acid pathways (e.g., glutamine, methionine, serine, and tryptophan) were also upregulated (Fig. 5E). In addition to amino acid biosynthesis, the OppABCDF machinery, which imports tripeptides from the environment (55), was upregulated in response to FADDI-019. The OppABCDF machinery is important for quorum sensing and nutrition (55). Opp transporters have been shown to supply bacteria with exogenous peptides that serve as amino acid resources. The activation of the Opp transport system is therefore synonymous with amino acid production. The role of amino acid biosynthesis and peptide import in the response to CAMPs is currently not well understood. However, in response to FADDI-019 treatment, we observed the strongest activation of gene expression, compared to all other differentially expressed genes, in amino acid biosynthesis genes. Taken together, we propose that the increase in vancomycin resistance gene expression and cell wall metabolism drives the need for increased amino acid production and import of tripeptides.

FADDI-019 also significantly repressed genes involved in the virulence of *S. aureus*. The repression of *saeRS* and the SaeRS-regulated virulence factor *sbi* by other, alpha-helical CAMPs, i.e., ovisporin-1, temporin L, and dermaseptin K4-S4, has been shown previously (21, 22). However, other known SaeRS-regulated genes (e.g., *coa*, *fnbB*, and *saeQP*), which were similarly repressed in our study, were not repressed by those alpha-helical CAMPs in *S. aureus* ATCC 25904 (21, 22). These differences are most likely due to differences in the mode of action between FADDI-019 and alpha-helical CAMPs and/or biological variability between *S. aureus* strains. In addition to the repression of SaeRS-regulated genes, four enterotoxin genes were also dramatically repressed by FADDI-019 treatment. Interestingly, this response is also specific to FADDI-019 and has not been reported for other CAMPs. Our data suggest that, in addition to killing *S. aureus* ATCC 700699, it is very likely that FADDI-019 reduced the virulence of the infecting bacterial cells as a secondary effect. When making comparisons between strains, it is important to note that the strain used in this study is a vancomycin-intermediate *S. aureus* (VISA), whereas *S. aureus* ATCC 25904 is vancomycin susceptible. VISA strains have been shown to have mutations in regulatory genes, including *vraSR*, which alter the transcription profile and result in intermediate vancomycin resistance (56, 57). The SaeSR two-component system is not known to be differentially regulated in different VISA strains (56).

Our transcriptomic analysis of *S. aureus* ATCC 700699 during FADDI-019 exposure demonstrated that a large number of essential metabolism processes, including folate biosynthesis, were upregulated (Fig. 5D). Some of these changes have been previously observed with alpha-helical CAMPs (22); however, the upregulation of folate biosynthesis genes was not reported with alpha-helical-CAMP treatment in that study. We hypothesized that these perturbations are important for *S. aureus* ATCC 700699 during FADDI-019 treatment. Pathway analysis (Fig. 5B) revealed that tetrahydrofolate production would be a suitable chokepoint target for a synergistic combination with FADDI-019. Indeed, as a test of this hypothesis, we subsequently demonstrated that FADDI-019 and sulfamethoxazole in combination were synergistic, with substantial reductions in the MICs of both compounds (Table 2). Our systems approach, exemplified here, holds much promise for identifying novel approaches to identify rational antimicrobial combinations in the future.

In conclusion, in an era of burgeoning multidrug resistance and diminishing therapeutic options, it is crucial that every effort must be made to discover antibacterial compounds against problematic pathogens. This is the first study reporting the transcriptomics of an unexpected antibacterial activity of a novel synthetic polymyxin analog against Gram-positive *S. aureus*. Importantly, our systems approach using transcriptomics has shown that pathway analysis of responses to drugs has a significant potential in predicting synergistic antibiotic combinations.

## MATERIALS AND METHODS

### Bacterial strains, culturing conditions, and susceptibility tests.

*S. aureus* ATCC 700699 (Mu50, VISA), *S. aureus* ATCC 700698 (Mu3, heterogeneous VISA), *A. baumannii* ATCC 17978, *A. baumannii* ATCC 19606, and *P. aeruginosa* ATCC 27853 were grown at 37°C on Mueller-Hinton (MH) agar plates or in cation-adjusted Mueller-Hinton broth (CAMHB) (Ca^2+^ at 22.5 mg/liter and Mg^2+^ at 11.25 mg/liter). No antibiotic selection was used unless stated otherwise. The MICs of FADDI-019, colistin (Sigma-Aldrich, Castle Hill, Australia), polymyxin B (Sigma-Aldrich), and sulfamethoxazole (Sigma-Aldrich) were determined by broth microdilution in CAMHB according to the Clinical and Laboratory Standards Institute protocol (*n* = 3) (63). The synergistic effect by FADDI-019 with sulfamethoxazole was examined using a checkerboard method (see Table S2 in the supplemental material) (58).

10.1128/mSphere.00119-16.2 Table S2  Checkerboard analysis of sulfamethoxazole and FADDI-019 combination therapy. Download Table S2, PDF file, 0.04 MB. Copyright © 2016 Zhao et al. 2016 Zhao et al. This content is distributed under the terms of the Creative Commons Attribution 4.0 International license.

### Time-kill studies.

The time-kill kinetics of FADDI-019 against *S. aureus* ATCC 700699 was examined in three replicates at 2×, 4×, and 8× MIC with a log-phase broth culture (optical density at 600 nm [OD_600_] of 0.4, 10^7^ CFU/ml) (59). Viable bacteria were enumerated on nutrient agar plates from samples collected at 0, 0.5, 1, 2, 4, 6, 10, and 24 h after treatment. Colonies were counted following overnight incubation at 37°C, and the lower limit of detection was 20 CFU/ml.

### Preparation of cells for SEM.

Cultures of *S. aureus* ATCC 700699 were incubated at 37°C with aeration until the OD_600_ reached 0.4. FADDI-019 was added to a final concentration of 1×, 4×, or 8× MIC, and cultures were incubated for an hour at 37°C. An untreated sample and a sample treated with 99.5 mg/liter polymyxin B were used as controls. After the incubation, cells were pelleted by centrifugation at 5,000 × *g* for 10 min. Cells were fixed by the addition of 2.5% glutaraldehyde for 1 min and then washed three times using phosphate-buffered saline (PBS) (pH 7.4). The bacterial cultures were air dried onto polyethylenimine-coated coverslips and immersed for an hour in 2.5% glutaraldehyde in PBS. The slides were then washed with PBS three times, and dehydration was done using 10% increments of ethanol in water from 0 to 100% for 10 min at each step. The coverslips were dried using a Balzers critical point dryer (Balzers, Liechtenstein, Germany) prior to mounting on 20-mm aluminum stubs with double-sided carbon tabs. The cells were coated with gold and imaged with a Philips XL30 field-emission scanning electron microscope (SEM; Philips, Eindhoven, Holland) at the University of Melbourne (Victoria, Australia).

### Flow cytometry.

Cultures (10 ml) of *S. aureus* ATCC 700699 were incubated at 37°C until the OD_600_ reached 0.4 and then treated with either polymyxin B (99.5 mg/liter) or FADDI-019 at 4× MIC. Samples (300 µl) were taken from each culture at 0, 15, and 60 min after treatment and incubated for 10 min with DiOC_2_(3) (3,3'-diethyloxacarbocyanine iodide; Thermo Fisher, Victoria, Australia). Samples (300 µl) were analyzed by flow cytometry (NovoCyte; ACEA Biosciences, Inc.) with the laser set to emit at 488 nm, and fluorescence was measured in the red and green channels. All incidents were plotted on a two-dimensional (2-D) scatterplot using their respective fluorescence intensities in each channel.

### RNA-Seq transcriptomics.

*S. aureus* ATCC 700699 was grown to an OD_600_ of 0.4 from an initial absorbance of 0.005, and FADDI-019 was added to a final concentration of 4× MIC. Samples were collected at 0, 15, and 60 min posttreatment and preserved with RNAprotect (Qiagen, United States) following the manufacturer’s instructions. Cells were pelleted by centrifugation at 5,000 × *g* for 10 min at 4°C. RNA was isolated using an RNeasy minikit (Qiagen) in accordance with the manufacturer’s instructions, with the following additions. Cell pellets were homogenized in 1 ml Tris-buffered saline (TBS) (20 mM Tris, pH 7.5) containing 0.4 mg of lysostaphin and incubated at 37°C for 15 min. Subsequently, 20 mg of lysozyme in TE buffer (20 mM Tris, pH 7.5, 2 mM EDTA, pH 7.8) was added and the sample was incubated at 25°C for 10 min. Control samples were collected from an antibiotic-free culture, and each experiment was repeated three times. RNA sequencing was conducted by the MHTP High-Throughput Sequencing Facility at the Hudson Institute of Medical Research (Clayton, Victoria, Australia).

### Bioinformatic analysis.

RNA sequence reads were independently aligned with the genome sequence of *S. aureus* ATCC 700699 (NCBI accession number NC_002758.2) using Subread (Victorian Bioinformatics Consortium, http://www.vicbioinformatics.com/software.shtml) (60, 61). The RNA sequence data from biological replicates were analyzed using the voom and limma linear modeling methods via the Degust interactive Web-based RNA-seq visualization software (http://www.vicbioinformatics.com/degust/). Differentially expressed genes were defined as those with a change in expression of >2-fold and a corresponding false discovery rate (FDR) of <0.05. The gene ontology terms and pathways were annotated via BioCyc and KEGG. Principal component analysis was performed using the R statistical computing package (62). For pathway analysis, genes shown to be differentially expressed at 15 min after treatment relative to their expression in the control were mapped onto the metabolic network of *S. aureus* ATCC 700699 (biocyc.org). Pathways containing multiple differentially expressed genes were selected for chokepoint analysis (biocyc.org).
