# Supplementary material for: Transcriptomic Analysis of the Activity of a Novel Polymyxin against Staphylococcus aureus
Source: mSphere. 2016 Jul 27;1(4):e00119-16. doi: 10.1128/mSphere.00119-16 (PMC4963539; doi:10.1128/mSphere.00119-16)
Supplement: Table S1 [file sph004162117st1.pdf]

Supplementary Table S1: Full list of differentially regulated genes with their respective expression ratio expressed in log2, false discovery rate (FDR), annotation, and pathway.

Gene	Expression ratio(log_2)	FDR	Protein/ortholog	Pathway
butA	1.71	2.36E-14	Acetoin reductase	Butanoate metabolism
metE	2.23	3.03E-13	5-methyltetrahydropteroyltriglutamate- Homocysteine methyltransferase	Cysteine and methionine metabolism
gltB	1.92	4.08E-16	Glutamate synthase large subunit	Alanine, aspartate and glutamate metabolism
gltD	1.84	1.10E-14	NADH-glutamate synthase small subunit	Nitrogen metabolism
oppB	1.65	3.58E-13	Oligopeptide transport system permease protein	Beta-Lactam resistance
oppC	1.73	1.21E-12	Oligopeptide transport system permease protein	ABC transporters
oppD	1.70	6.86E-14	Oligopeptide transport system ATP-binding protein	
oppF	1.64	1.73E-12	Oligopeptide transport system ATP-binding protein	
oppA	1.67	1.04E-13	Similar to peptide binding protein	
dhoM	2.63	2.94E-14	Homoserine dehydrogenase	Glycine, serine and threonine metabolism
thrC	2.17	3.24E-13	Threonine synthase	
thrB	2.27	7.45E-14	Homoserine kinase homolog	
citC	1.99	2.12E-14	Isocitrate dehyrogenase	Citrate cycle
citZ	2.01	4.13E-15	Citrate synthase II	Glutathione metabolism
serA	1.67	1.38E-11	D-3-phosphoglycerate dehydrogenase	Glycine, serine and threonine metabolism
vraS	2.33	6.29E-14	Two-component sensor histidine kinase	Two-component system
vraR	2.29	9.77E-14	Two-component response regulator	
ilvD	3.49	7.03E-13	Dihydroxy-acid dehydratase	Valine, leucine and isoleucine biosynthesis
ilvB	3.33	1.63E-13	Acetolactate synthase large subunit	
ilvC	5.21	6.60E-14	Alpha-keto-beta-hydroxylacil reductoisomerase	
leuA	4.53	9.77E-14	2-isopropylmalate synthase	
leuB	4.30	5.27E-14	3-isopropylmalate dehydrogenase	
leuC	4.02	1.66E-14	3-isopropylmalate dehydratase large subunit	
leuD	3.71	5.62E-13	3-isopropylmalate dehydratase small subunit	
ilvA	3.17	9.56E-14	Thereonine dehydratase	
fbp	1.79	9.86E-14	Fructose-bisphosphatase	Glycolysis /Gluconeogenesis
geh	-1.68	9.56E-14	Glycerol ester hydrolase	Triacylglycerol degradation
saeS	-1.96	2.33E-16	Histidine protein kinase	Two-component system
saeR	-2.01	4.13E-15	Response regulator	
saeP	-2.07	1.06E-13	Hypothetical protein	
saeQ	-3.15	5.93E-17	Hypothetical protein	
qoxC	-1.73	5.24E-15	Quinol oxidase polypeptide III	Oxidative phosphorylation
qoxB	-1.87	9.77E-14	Quinol oxidase polypeptide I	
qoxD	-1.75	2.98E-12	Putative quinol oxidase polypeptide IV QoxD	
sbi	-2.77	9.85E-14	IgG-binding protein	Staphylococcus aureus infection
ispD	1.72	3.82E-10	Similar to 2-C-methyl-D-erythritol 4-phosphate cytidylyltransferase	Terpenoid backbone biosynthesis
prfB	2.45	3.76E-12	Peptide chain release factor 2	Macromolecule metabolism
pgi	1.06	4.26E-13	Glucose-6-phosphate isomerase A	Glycolysis / Gluconeogenesis
folD	1.10	1.68E-11	FolD bifunctional protein	One carbon pool by folate<br>Metabolic pathways<br>Microbial metabolism in diverse environments<br>Carbon metabolism
katA	1.22	5.13E-10	Catalase	Tryptophan metabolism
rmpG	1.11	6.91E-08	50S ribosomal protein L33	Ribosome
rpsN	1.06	1.05E-11	30S ribosomal protein S14 homolog	
trpC	2.00	2.26E-08	Indole-3-glycerol phosphate synthase	Tryptophan biosynthesis
trpF	1.87	9.38E-07	Phosphoriborylanthranilate isomerase	
trpB	1.60	3.05E-07	Tryptophan synthase beta chain	
pstB	1.53	4.93E-09	Phosphate ABC transporter	ABC transporters
ctpA	1.49	3.21E-14	Probable carboxy-terminal processing proteinase	ABC transporters
malA	1.48	6.17E-12	Alpha-D-1,4-glucosidase	Galactose metabolism
malR	1.27	2.05E-10	Maltose operon transcriptional repressor	Starch and sucrose metabolism
fhs	1.30	9.56E-14	Formyltetrahydrofolate synthetase	One carbon pool by folate
prsA	1.77	1.11E-13	Peptidyl-prolyl cis/trans isomerase	
arg	1.32	5.62E-11	Arginase	Arginine and proline metabolism
lacE	1.07	3.00E-09	PTS system lactose-specific IIBC component PTS system	Galactose metabolism
lacF	1.19	1.83E-06	lactose-specific IIA component	Phosphotransferase system (PTS)
lacD	1.13	3.85E-08	Tagatose 1,6-diphosphate aldolase	
lacC	1.25	4.60E-08	Tagatose-6-phosphate kinase	
rocA	1.81	4.47E-11	1-pyrroline-5-carboxylate dehydrogenase	Alanine, aspartate and glutamate metabolism<br>Arginine and proline metabolism
isaB	1.33	3.80E-11	Immunodominant antigen B	
coa	-1.19	4.95E-13	Amino acid permease	Triacylglycerol degradation
rpsF	-1.23	4.26E-08	30S ribosomal protein S6	Ribosome
ssb	-1.28	7.11E-11	Single-strand DNA-binding protein of phage phi PVL<br>30S ribosomal protein S18	DNA replication
rpsR	-1.31	4.95E-13		Mismatch repair<br>Homologous recombination
nupC	-1.32	9.77E-13	Pyrimidine nucleoside transport protein	

rplA	-1.09	1.71E-10	50S ribosomal protein L1	Ribosome
rplJ	-1.41	4.92E-15	50S ribosomal protein L10	
rplL	-1.33	7.28E-12	50S ribosomal protein L7/L12	
sdrE	-1.22	9.94E-13	Ser-Asp rich fibrinogen-binding, bone sialoprotein-binding protein	Staphylococcus aureus infection
nuc	-2.17	2.22E-12	Staphylococcal nuclease	Staphylococcal nuclease
potA	-1.51	8.55E-12	Spermidine/putrescine ABC transporter	ABC transporters
potB	-1.57	6.75E-13	Spermidine/putrescine ABC transporter	
potC	-1.49	4.93E-12	Spermidine/putrescine ABC transporter	
potD	-1.35	3.24E-13	Spermidine/putrescine-binding protein precursor	
rpsP	-1.21	6.21E-11	30S ribosomal protein S16	Ribosome
rimM	-1.26	3.94E-12	Probable 16S rRNA processing protein	Ribosome
trmD	-1.28	1.04E-13	tRNA-mehtyltransferase	
rplS	-1.59	7.84E-12	50S ribosomal protein L19	Ribosome
rpmA	-1.08	8.07E-13	50S ribosomal protei L27	Ribosome
rplT	-1.25	2.15E-12	50S ribosomal protein L20	Ribosome
infC	-1.12	4.41E-12	Translation initiation factor IF-3	
ThiI	-1.13	1.43E-10	Probable thiamine biosynthesis protein	Thiamine metabolism
rpsD	-1.31	5.35E-12	30S ribosomal protein S4	Ribosome
yent2	-1.06	6.89E-05	Enterotoxin	Staphylococcus aureus infection
sei	-1.42	5.43E-08	Enterotoxin	
sem	-1.13	3.14E-06	Enterotoxin	
seo	-1.55	2.72E-10	Enterotoxin	
tnp	-1.70	6.02E-05	Transposase	
hlb	-1.66	2.82E-13	Truncated beta-hemolysin	Defence mechanisms<br>Virulence factors
czrA	-1.67	1.71E-09	Repressor protein	
czrB	-1.49	2.85E-10	Cation-efflux system membrane protein	
rpsl	-1.73	1.33E-12	30S ribosomal protein S9	Ribosome
rplM	-1.60	4.73E-10	50S ribosomal protein L13	
rplF	-1.16	1.53E-09	50S ribosomal protein L6	
fnbB	-1.37	9.08E-12	Fibronectin-binding protein	Bacterial invasion of epithelial cells
