# Supplementary material for: Transcriptomic Analysis of the Activity of a Novel Polymyxin against Staphylococcus aureus
Source: mSphere. 2016 Jul 27;1(4):e00119-16. doi: 10.1128/mSphere.00119-16 (PMC4963539; doi:10.1128/mSphere.00119-16)
Supplement: Table S2 [file sph004162117st2.pdf]

Table S2. MIC checkerboard test of sulfamethoxazol and FADDI-019 in combination against *S. aureus* ATCC 700699.

Sulfamethoxazol

FADDI-019

**mg/L**	**512**	**256**	**128**	**64**	**32**	**16**	**8**	**4**	**2**	**1**	**0**	**Blank**
**128**	-	-	-	-	-	-	-	-	-	-	-	-
**64**	-	-	-	-	-	-	-	-	-	-	-	-
**32**	-	-	-	-	-	-	-	-	-	-	-	-
**16**	-	-	-	-	-	-	-	-	-	-	-	-
**8**	-	-	-	-	-	-	+	++	++	++	++	-
**4**	-	-	-	-	-	-	+	+	++	+	++	-
**2**	-	-	-	++	++	+	+++	+++	+++	+++	+++	-
**0**	-	-	-	++	+++	+++	+++	+++	+++	+++	+++	-

- OD^{620} <0.005; + OD^{620} 0.05-0.2; ++ OD^{620} 0.2-0.5; +++ OD^{620} >0.5.
